# Massive hydropyonephrosis on pediatric patient: A case report of management and source control

**DOI:** 10.1016/j.ijscr.2024.110766

**Published:** 2024-12-23

**Authors:** Nadya Rahmatika, Soetojo Wirjopranoto, Yufi Aulia Azmi, Antonius Galih Pranesdha Putra, Kevin Muliawan Soetanto

**Affiliations:** a Faculty of Medicine, Wijaya Kusuma University, Surabaya, Indonesia; b Department of Urology, Faculty of Medicine Universitas Airlangga, Dr. Soetomo General Academic Hospital, Surabaya, Indonesia; c Department of Health Sciences, University of Groningen, University Medical Center Groningen, Groningen, the Netherlands; d Department of Immunology, Faculty of Medicine Siriraj Hospital, Mahidol University, Bangkok, Thailand; e Department of Biomedical Science, Faculty of Medicine, Universitas Surabaya, Indonesia

**Keywords:** Pediatric, sepsis, Mortality, Case report, Infection

## Abstract

**Introduction and importance:**

Dilation and stretching of the collecting system of the kidney due to obstruction of urine flow is called hydronephrosis. This case may be accompanied by the presence of pus known as pyonephrosis. This case report reporting massive pyonephrosis in pediatrics related to management and source of control.

**Case presentation:**

A 10-year-old boy came in with the main complaints of high fever, decreased appetite, vomiting, and nausea. The examination showed left severe hydronephrosis (+) with a size of 14.59 × 6.9 × 9.2 cm. The patient underwent percutaneous nephrostomy (PCN) and showed pus production. From the antegrade pyelography (APG) during PCN, it was stenosis of the left ureteropelvic junction (UPJ). Empirical antibiotics were administered, followed by albumin transfusion. Antibiotics were changed on day 3 post-PCN when urine culture results showed *Staphylococcus aureus*. After successful improvement of the general condition and minimal pus production from PCN, the patient had a Double J Stent (DJ) and pyeloplasty on the left UPJ. The patient was discharged on day 4 after the left pyeloplasty.

**Clinical discussion:**

Management of UPJ Stenosis with massive hydronephrosis complications can be done in two stages with the first stage being the diversion of pus from the kidney, then followed by pyeloplasty management. Management is continued with nephrostomy or ureteral stent placement for urine diversion. Management of bacterial infections is adjusted according to culture results.

**Conclusion:**

Management of hydronephrosis with pyonephrosis as complications can be be carried out in two stages, pus diversion, then followed by the pyeloplasty.

## Highlights

• Flank pain is a very common problem, and it can be possible as an emergency condition.
• Flank pain is one sign of emergency surgery that can be caused by infection, trauma, inflammation or obstruction.
• Urinary diversion is necessary to avoid worsening general condition
• This is complex case and related to sepsis in pediatric, which involves multiple procedures.
• Source control surgery is needed to overcome the hydropyonephrosis condition.

## 1 Introduction

Hydronephrosis is defined as the dilation and distension of the kidney-collecting system of one or both kidneys due to blockage of distal urine flow to the renal pelvis. Hydronephrosis occurs in all age groups. The presentation can be acute or chronic, physiological (very common in pregnant women) pathological, unilateral, or bilateral. [1]. The prevalence of these cases varies. 2–2.5 % of children suffer from hydronephrosis, with a slightly higher prevalence in boys [2]. Another study found similar results suggesting that the frequency was higher in boys. Cases of congenital hydronephrosis have an incidence of 0.13–0.16 %, with infant cases making up about 25 % of all cases. [3]. Hydronephrosis is a disorder most commonly detected on prenatal ultrasound in about 1 % to 5 % of all pregnancies. [4]. In the process, hydronephrosis can be followed by pyonephrosis. “Push under pressure” is the definition of pyonephrosis, which is the presence of infected urine in a blocked urine collection system [5]. Patients with pyonephrosis are sporadic. [6].

The causes and presentation of cases vary among age groups. In newborns and children, structural abnormalities are the main cause. [1]. The etiology for pediatric hydronephrosis in frequency order includes transient or physiological hydronephrosis, ureteropelvic junction obstruction, vesicoureteral reflux, and urinary obstructive uropathy. [7]. Hydronephrosis, often thought of as a marker of congenital abnormalities of the kidneys and urinary tract, is the most commonly detected abnormality on prenatal ultrasound. [4]. This is different from cases of pyonephrosis, which usually appears in young adults who experience blockage of ureteropelvic junctions from extrinsic sources or obstruction of stones. [5].

In these cases, the key points for a quick diagnosis, an ideal therapeutic approach, and follow-up of hydronephrosis associated with anomalies in children are the severity of hydronephrosis. [8]. Surgery can be performed openly or laparoscopic. [9]. Asymptomatic cases require monitoring, while in patients with recurrent symptoms and impaired renal function, timely surgical intervention is indicated. Surgical procedures, such as pyeloplasty, can be used to treat obstructions at the ureteropelvic junction and improve urine drainage in cases where treatment is needed. [10]. Percutaneous drainage, retrograde ureter stenting, pyeloplasty in case of UPJ obstruction, stone management for urolithiasis, and nephrectomy are some of the treatments available for pyonephrosis. [11].

Since asymptomatic hydronephrosis can resolve itself without treatment, there is no universally accepted method for managing it. The question of whether early surgical intervention is required to prevent renal function deterioration is still up for debate [12]. The management of hydronephrosis followed by pyonephrosis varies and is determined by the patient's condition. The key to managing this case has not been fully reported even though it can prevent a poor prognosis, especially in massive pyonephrosis case. This case report was prepared with the aim of reporting massive pyonephrosis in pediatrics related to management and control sources. Reports of this case have been reported in line with the SCARE Guidelines [13].

## 2 Case presentation

**Fig. 1:**
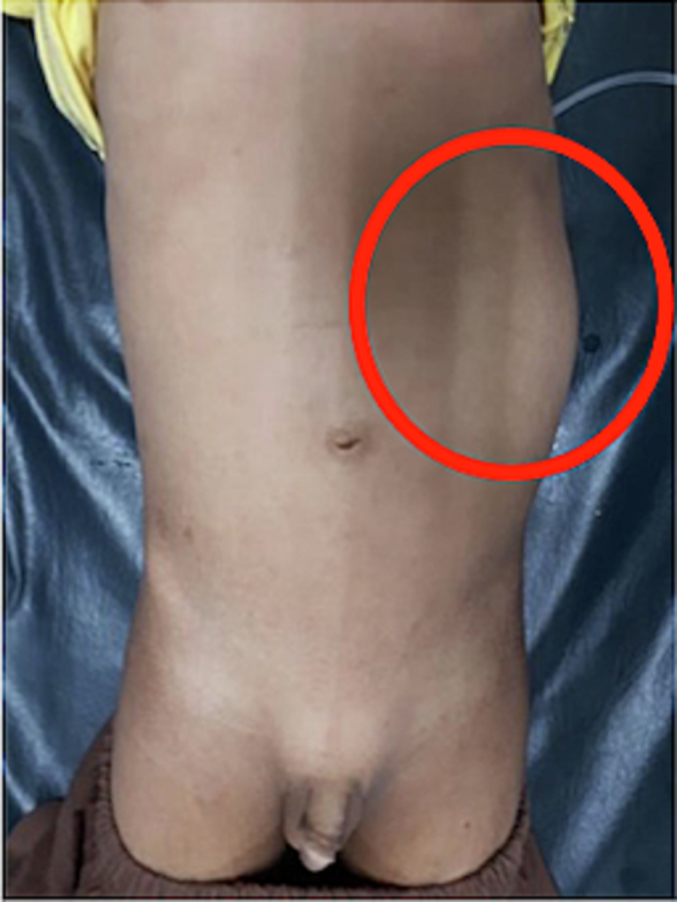
Clinical picture of the patient.

**Fig. 2:**
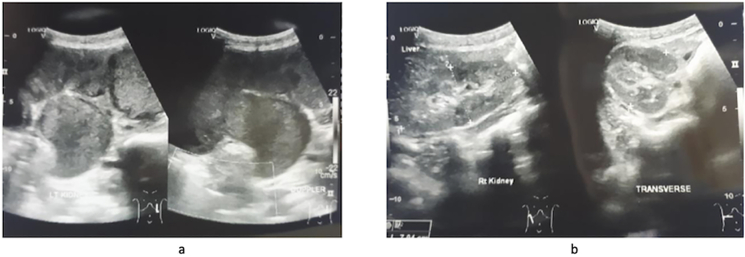
a. USG of the left kidney b. USG of the right kidney.

A boy 10 years old has had a chief complaint of a high fever for 2 days. The patient came to the emergency room with a high fever, loss of appetite since one week before admission, vomiting (+), and nausea (+). The Visual Analog Score (VAS) was 4. The physical examination showed GCS-456, HR −126 times/min, RR-26 times/min, Temperature-39.5C. Physical Examination showed weight-35 kg, height-145 cm, bowel sound (+) normal, and mass at the left upper left until lower quadrant, size 6 × 9 × 13 cm, seems mobile and cystic consistency (Fig. 1). Urine Production was 500 cc/12 h pyuria (+). Laboratory Examination Results showed hemoglobin-11 g/dL, leucocyte-45.92/uL, albumin-2.6, serum creatinine-0.63 mg/dL, and creatinine clearance-100 mL/min. An empirical antibiotic, ceftriaxone 1350 mg/24 h intravenous (iv), was given, followed by albumin 20 % transfusion in 24 h. Radiology Examination showed Ultrasonography (USG) was normal right kidney, left severe hydro-pyonephrosis (+) with size 14.59 × 6.9 × 9.2 cm accompanied with internal moving echo (Fig. 2). There was no medical history from the family with same disease or others.

**Fig. 3:**
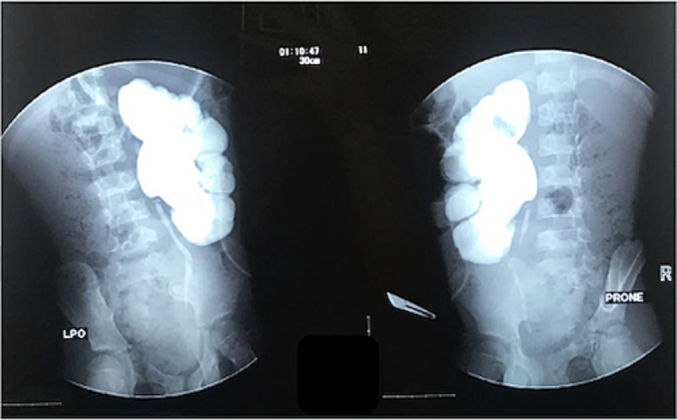
Left antegrade pyelography (APG).

The patient underwent percutaneous nephrostomy (PCN) with a pigtail size of 4.7 fr, which resulted in 550 cc of pus production in 24 h. Antegrade pyelography (APG) performed during PCN showed left ureteropelvic junction (UPJ) stenosis (Fig. 3). We changed the antibiotic with Ciprofloxacin 700 mg/12 h IV on day 3 post-PCN when the urine and blood culture results showed *Staphylococcus aureus*. Cultures were performed to ensure no *staphylococcal* infection before pyeloplasty and DJ stent placement. After successful general condition improvement and minimal pus production from PCN on day 6th after PCN, We performed DJ Stent insertion and dismembered-pyeloplasty Anderson Haynes technique on the left UPJ. Blood and urine evaluation results were normal before the patient went home.

The patient was discharged on day 4 after the left pyeloplasty. He came to the urology policlinic 1 week after discharge with no complaint, and the DJ Stent was removed 3 months after pyeloplasty. Routine 3-month policlinic visits in 1 year after pyeloplasty showed normal creatinine levels 0.5 mg/dL without any complaints.

## 3 Discussion

The risk factors present in this case that lead to the occurrence of hydropyonephrosis are being male. The prevalence of these cases varies. 2–2.5 % of children suffer from hydronephrosis, with a slightly higher prevalence in boys [2]. Another study found similar results suggesting that the frequency was higher in boys. Cases of congenital hydronephrosis have an incidence of 0.13–0.16 %, with infant cases making up about 25 % of all cases. [3]. In this study, the patient had no family history of the disease. Previous studies have found that one of the risks of hydronephrosis is diabetes. [12]. Diabetes mellitus, non-functioning kidneys upon admission, staghorn kidney stones, and moderate to severe hydronephrosis are some of the factors that may affect the development of pyonephrosis. Gender, history of urinary tract infection, chronic kidney disease, renal anatomic abnormalities, prior urologic treatments, positive urine culture for bacteremia, number of stones, and large size of kidney stones were additional characteristics that demonstrated statistical significance. Regarding long-term consequences, patients may pass away due to pyonephrosis-induced urosepsis. [14].

After a medical history review, the patient was identified as having fever, loss of appetite, vomiting, and nausea. Other studies have found fever, chills, and flank pain as the main complaints. [15]. Management of hydropyonephrosis cases in pediatrics is carried out with rapid diagnosis, therapeutic approach, and appropriate follow-up. Management of patients can be done in two stages with the first stage being the diversion of pus from the kidney, followed by pyeloplasty management. Management is continued with nephrostomy or ureteral stent placement for urine diversion. Management of bacterial infections is adjusted according to culture results. Another case report found a ten-year-old boy who presents with abdominal distension and renal failure has bilateral gigantic hydronephrosis brought on by obstruction of the pelvic ureteric junction. After computed CT verified the diagnosis, a two-stage treatment was necessary to restore kidney function: first, a percutaneous nephrostomy, and then an Anderson-Hynes pyeloplasty [16]. Anderson-Hynes pyeloplasty (AHP), is a surgical technique used to treat ureteral obstruction, or pelvic-ureteral junction obstruction (PUJO). The procedure involves dissection, incision, spatulation, anastomosis, and repair. AHP was open pyeloneplasty [17]. Other cases of giant hydronephrosis can be treated with just a nephrectomy and the patient was in good health. [18].

Patients in this study underwent Anderson Haynes technique pyeloplasty on the left UPJ. Pyeloplasty is considered the gold standard for the treatment of obstruction in UPJ and can be divided into incision and flap procedures. Dissected pyeloplasty, also referred to as Anderson-Hynes surgery, is a versatile surgical technique characterized by total dissection of the ureter and removal of the affected segment. This procedure allows the correction of problems such as excessive pelvis and transposition of the UPJ in cases where the vessels that cross are blocking the flow of urine. Flap procedures such as the Foley Y-V plasty present benefits such as reduced operating time and a reduced risk of UPJ devascularization, making them suitable for addressing long ureteral constriction. In providing pyeloplasty management, tailoring the procedure options to the specific characteristics of each case ensures the best outcome for patients undergoing pyeloplasty [4]. Percutaneous drainage, retrograde ureteral stenting, pyeloplasty in the event of UPJ obstruction, stone management for urolithiasis, and nephrectomy are among the available treatments for pyonephrosis [11].

The patient was discharged on day 4 after the left pyeloplasty. He came to the urology policlinic 1 week after discharge with no complaint. And the DJ Stent was removed 3 months after pyeloplasty. Routine 3-month policlinic visits in 1 year after pyeloplasty showed normal creatinine levels 0.5 mg/dL without any complaints.

Children with hydronephrosis suspected obstruction and normal renal parenchyma may be followed for at least 2 years to allow for spontaneous resolution before being referred to urology. [19]. Although many patients with hydronephrosis experience spontaneous improvement, some may experience symptomatic problems, such as UTIs. Although often asymptomatic and capable of spontaneous resolution, surgical intervention is necessary for certain scenarios such as febrile urinary tract infections, worsening hydronephrosis, or decreased kidney function. [4].

This patient underwent percutaneous nephrotomy (PCN) first. PCN is a minimally invasive temporary or permanent X-ray-guided procedural alternative to traditional surgery in patients with hydronephrosis. PCN is the procedure of choice when transurethral access is not possible or has failed to free the blocked urinary system from the effects of extrinsic masses (e.g., pregnancy, malignancy, fluid pools such as cysts, abscesses, or urinomas) or intrinsic blockages (e.g., benign or malignant strictures). Drainage of a blocked renal unit is the most common indication for PCN, accounting for 85 % to 90 % of all nephrotomy placements. Unless infected, draining a blocked hydronephrotic kidney is not an acute emergency. [20]. Another study found that double J stenting was a safer, faster, and more effective way to treat infective hydronephrosis and pyonephrosis than percutaneous nephrectomy with a lower incidence of complications. [21]. The patient in this case did not undergo retrograde stenting before PCN.

## 4 Conclusion

The management of UPJ Stenosis with complications of massive hydronephrosis with pyonephrosis as complication can be carried out in two stages, with the first stage of pus diversion from the kidney, in this case, using PCN, followed by the definitive management, pyeloplasty. Treatment of *Staphylococcus aureus* infection requires proper effort due to the risk of widespread antibiotic resistance. In cases of hydronephrosis in children, rapid diagnostics, therapeutic approaches, and appropriate follow-up are managed.

## Registration of research studies

1. Name of the registry: Not required
2. Unique identifying number or registration ID: Not required
3. Hyperlink to your specific registration (must be publicly accessible and will be checked): Not required

## Additional information

All information is private for this paper.

## Consent

Written informed consent was obtained from the patient's parents/legal guardian for publication and any accompanying images. A copy of the written consent is available for review by the Editor-in-Chief of this journal on request.

## Ethical approval

Ethical approval has been approved for this case report.

Ethical approval for this study was provided by Health Research Ethics Committee of Dr. Soetomo General-Academic Hospital, Surabaya.

## Guarantor

Soetojo Wirjopranoto

## Author contributions

Nadya Rahmatika: Conceptualization, Data Curation, Writing-Original draft preparation.

Soetojo Wirjopranoto: Conceptualization, Methodology, Data Curation, Investigation, Writing-Original draft preparation, Supervision, Validation.

Yufi Aulia Azmi: Conceptualization, Methodology, Data Curation, Investigation, Writing-Original draft preparation, Supervision, Validation.

Antonius Galih Pranesdha Putra: Writing-Original draft preparation, Writing-Reviewing, and Editing.

Kevin Muliawan Soetanto: Writing-Original draft preparation, Writing-Reviewing, and Editing.

## Funding statement

The author(s) received no financial support for the research.

## Declaration of competing interest

The authors declare no conflict of interest.

## Data Availability

No data was used for the research described in the article.
